# Association between baseline insulin resistance and psoriasis incidence: the Women’s Health Initiative

**DOI:** 10.1007/s00403-021-02298-9

**Published:** 2021-11-24

**Authors:** Alfred A. Chan, Houmin Li, Wendy Li, Kathy Pan, Jennifer K. Yee, Rowan T. Chlebowski, Delphine J. Lee

**Affiliations:** 1 Division of Dermatology, The Lundquist Institute, 1124 West Carson Street, Torrance, CA 90503 USA; 2 Division of Dermatology, Peking University People’s Hospital, Xicheng District, Beijing, China; 3 Harbor-UCLA Medical Center, Torrance, CA USA; 4 Division of Medical Oncology and Hematology, The Lundquist Institute, Torrance, CA USA; 5 Division of Pediatric Endocrinology, The Lundquist Institute, Torrance, CA USA; 6 David Geffen School of Medicine, University of California Los Angeles, Los Angeles, CA USA

**Keywords:** Psoriasis, Insulin resistance

## Abstract

Small-scale studies offer conflicting evidence regarding the relationship/association between psoriasis and insulin resistance by HOMA-IR (homeostasis model assessment of insulin resistance). The purpose of this study was to assess the association between baseline HOMA-IR and psoriasis incidence in a large-scale longitudinal cohort of postmenopausal women. The analysis included 21,789 postmenopausal women from the Women’s Health Initiative. Psoriasis diagnosis was defined by fee-for-service Medicare ICD-9-CM codes assigned by dermatologists or rheumatologists, and a 2-year lookback period to exclude prevalent cases. Baseline HOMA-IR was calculated using the updated HOMA2 model. Hazard rates from the Cox regression models were stratified by age (10-year intervals), on WHI component (Clinical Trial or Observational Study), and on randomization status within each of the WHI clinical trials. The complete model also adjusted for ethnicity, waist–hip-ratio, and smoking and alcohol habits. Among participants free of psoriasis at entry, those with high baseline HOMA-IR (≥ 2) compared to low (< 1.4) had significantly higher risk for psoriasis over 21-year cumulative follow-up (HR: 1.39, 95% CI 1.08–1.79, *P*-trend: 0.011). In postmenopausal women, higher baseline HOMA-IR levels were significantly associated with higher incidence of psoriasis over 21-year cumulative follow-up. Results from this time-to-event analysis indicate that insulin resistance can precede and is associated with an increased risk of psoriasis. Study is limited by Medicare diagnostic code accuracy and cohort age.

**Supplementary Information:**

The online version contains supplementary material available at 10.1007/s00403-021-02298-9.

## Introduction

Psoriasis is a chronic inflammatory skin disease that affects about 2–4% of the U.S. population. Psoriasis can occur at any age, but peaks between age 20 and 30 years and between 50 and 60 years [1]. In postmenopausal women, the fall in estrogen concentration has been attributed to exacerbation of psoriasis [2]. According to a survey initiated by the National Psoriasis Foundation, 94% of patients reported that psoriasis interferes with their quality of life on a daily basis [3]. Although psoriasis has been traditionally regarded as a disease limited to the skin, it is now well known that it has important health implications beyond the skin [4].

Psoriasis is associated with significant comorbidities including type 2 diabetes [5]. The current evidence hints at a complex relationship between psoriasis and insulin resistance. Small case–control studies (*n* < 200 participants) have attempted to characterize the relationship between psoriasis and insulin resistance using the homeostasis model assessment of insulin resistance (HOMA-IR), a reliable and validated surrogate marker of insulin resistance [6–8]. However, the results have been conflicting. For example, Dhara et al. reported significantly higher HOMA-IR in psoriasis patients compared to age and sex-matched controls [9]. While Pereira et al. found that psoriasis patients are more than twice as likely to exhibit impaired glucose metabolism than controls, HOMA-IR did not significantly differ between the two groups in those with normal glucose tolerance [10]. Gyldenløve reported a significant association between psoriasis and insulin resistance when assessing with the hyperinsulinemic euglycemic clamp (HEC), but not with HOMA-IR [11].

We propose to better characterize the relationship between psoriasis and insulin resistance (via HOMA-IR) in a large cohort of postmenopausal women. Previous case–control studies have reported increased incidence of diabetes in psoriasis patients [12, 13]. Our study instead aims to explore the reverse: whether a pre-diabetic condition such as high baseline insulin resistance is a predictor of psoriasis. To our knowledge, this is the first study using a large-scale longitudinal cohort to investigate the association between baseline insulin resistance and psoriasis incidence in postmenopausal women.

## Patients and methods

### Study population

The Women’s Health Initiative (WHI) recruited postmenopausal women ages 50–79 years from across 40 US clinical centers between 1993 and 1998. This includes a “Clinical Trial” cohort (n = 68,132) with the following components: Estrogen-alone trial, Estrogen-plus-Progestin trial, Dietary Modification trial, and Calcium and Vitamin D trial. Each randomized controlled trial has its own exclusionary criteria involving safety, adherence, and retention concerns. Women ineligible or unwilling to join the clinical trials were invited to join the “Observational Study” cohort (*n* = 93,676). Detailed eligibility criteria and recruitment methods have been previously published [14]. Human subjects review committees at all participating sites approved WHI protocols and participants provided written informed consent.

Of the starting 161,808 postmenopausal women, 31,897 participants had at least one blood draw at enrollment measuring both insulin and glucose for HOMA-IR calculations. Of those, 23,093 women were linked to Medicare fee-for-service Parts A and B (FFS A + B). The final analytic cohort (*n* = 21,789) excluded 1,304 women who had prevalent cases of psoriasis or were not followed long enough for a 2-year lookback period.

### Data collection

At baseline, information on demographics, medical history, and lifestyle behaviors (such as smoking, alcohol, and exercise habits) were obtained through a self-administered questionnaire. Data on lifetime hormone use were obtained by a trained interviewer, assisted by charts displaying colored photographs of various hormone preparations. Trained staff also obtained anthropometric measurements such as height, weight, and waist and hip circumferences. The total Metabolic Equivalent of Task (MET-hours per week) was calculated by multiplying the MET levels for activity by the hours exercised per week and summing the values for all activities.

### Psoriasis outcome ascertainment

We classified subjects with psoriasis as previously described [15]. Briefly, psoriasis was defined by fee-for-service Medicare claims using the International Classification of Diseases, Ninth Revision, Clinical Modification (ICD-9-CM) diagnosis codes 696.0 (psoriatic arthropathy) and 696.1 (other psoriasis). To increase the validity of identifying individuals with psoriatic disease, the designation was limited to ICD-9-CM codes given by a dermatologist or rheumatologist. In a study of a managed care patient population in Northern California, psoriasis ICD-9-CM codes reported specifically by a dermatologist have a positive predictive value of 89% (95% CI, 79–95%) [16]. In addition, a 2-year lookback or washout period was implemented so as to not misclassify prevalent psoriasis cases as incidence [15].

### Determination of HOMA-IR

Baseline blood draws (Year 0) were excluded if they were drawn after less than 12 h of fasting. Glucose was analyzed using the hexokinase method. Fasting insulin was analyzed by the following methods and detection systems: BMD ES3000 Immunoassay System, Roche 2010 Electrochemiluminescence, Radioimmunoassay (linco Research, St. Louis, MO), and Sandwich Immunoassay (Roche Diagnostics). The analytes were similarly distributed across the various testing methods; much of the differences could be attributed to the demographics selected for the ancillary studies (Figure S1). Insulin resistance was calculated using the HOMA2 version 2.2.3, which is an updated HOMA computer model with nonlinear solutions that account for both circulating proinsulin and variations in hepatic and peripheral glucose resistance; acceptable steady-state input values were 20 to 400 pmol/L for insulin and 3.0 to 25.0 mmol/L for glucose [17]. Degree of insulin resistance was categorized as defined by previous studies: Low (HOMA-IR < 1.4), Moderate (1.4 ≤ HOMA-IR < 2.0), and High (HOMA-IR ≥ 2.0) [18–20].

### Statistical analysis

For primary analyses, we used time-to-event Cox proportional hazards regressions to estimate hazard ratios (HRs) and 95% confidence intervals (CIs) to study the association between baseline HOMA-IR and psoriasis incidence over the cumulative 21-year follow-up. Participants entered the risk set upon completion of the 2-year lookback period and a delayed-entry was applied for those who entered the risk set after WHI randomization. Event times were censored at the date of first psoriasis diagnosis, date no longer enrolled in FFS A + B, death or date of last follow-up through June 2017, whichever came first.

Hazard rates during follow-up were stratified on age (10-year intervals), on WHI component (Clinical Trial or Observational Study), and on randomization status within each of the WHI clinical trials (Diet Modification, Hormone Therapy, Calcium and Vitamin-D Trial). In addition, the following baseline characteristics were included in the Cox regression model to control for potential confounding effects for psoriasis based on previous literature and based on our univariable analyses: ethnicity (Caucasian, Asian, African-American, Hispanic, Other/Unspecified), continuous waist–hip-ratios, smoking (non-smoker, past-smoker, current-smoker), and alcohol habits (non-drinker, past-drinker, current drinker) [21, 22] (Table 2). Separate models with and without adjustment for these covariates were developed and compared. The proportional hazards assumption was tested with Schoenfeld residuals, and no violation of the proportionality assumption was found. In secondary analyses, we tested for interaction between baseline HOMA-IR and smoking habit on psoriasis.

## Results

HOMA-IR showed a weak positive correlation with baseline BMI (*R* = 0.46) and WHR (*R* = 0.34). Figure S2 shows a subgroup of women who were obese (BMI > 30 kg/m^2^ or WHR > 0.85), but had low insulin resistance and vice versa. Baseline characteristics by HOMA-IR are described in Table 1. Women with high HOMA-IR (≥ 2.0) were more likely to be randomized into the WHI Dietary Modification trial, but less likely to the Estrogen-plus-Progestin trial. Those with high HOMA-IR were also more likely to be younger (50–59 years), African-American, obese, prior alcohol drinkers, prior smokers, with prior hysterectomy, with fewer years of education, or fewer hours of physical activity. They were more likely to have a history of diabetes, hypertension, cardiovascular disease, stroke, liver disease, or rheumatoid arthritis. On the other hand, low HOMA-IR was associated with non-melanoma skin cancer and with the use of estrogen or estrogen-plus-progestin at baseline.

**Table 1:** Baseline characteristics by HOMA-IR

		Low HOMA-IR *n* (%)	Moderate HOMA-IR *n* (%)	High HOMA-IR n (%)	*P* value
Clinical trials					
Estrogen-Alone Trial					< 0.001
	Not randomized	7832 (66.4%)	1977 (16.8%)	1990 (16.9%)	
	Placebo	1277 (59.9%)	424 (19.9%)	430 (20.2%)	
	Treated	1263 (61.4%)	367 (17.9%)	426 (20.7%)	
Estrogen + Progestin Trial					< 0.001
	Not randomized	7832 (66.4%)	1977 (16.8%)	1990 (16.9%)	
	Placebo	2052 (72.3%)	446 (15.7%)	342 (12.0%)	
	Treated	2060 (69.5%)	487 (16.4%)	416 (14.0%)	
Calcium Vitamin D Trial					0.496
	Not randomized	9579 (66.6%)	2400 (16.7%)	2399 (16.7%)	
	Placebo	2439 (65.9%)	649 (17.5%)	612 (16.5%)	
	Treated	2466 (66.5%)	652 (17.6%)	593 (16.0%)	
Dietary Modification Trial					< 0.001
	Not randomized	10,834 (69.2%)	2465 (15.7%)	2367 (15.1%)	
	Placebo	1506 (60.8%)	503 (20.3%)	466 (18.8%)	
	Treated	2144 (58.8%)	733 (20.1%)	771 (21.1%)	
Demographics					
Age					< 0.001
	50–59	3601 (62.8%)	1027 (17.9%)	1105 (19.3%)	
	60–69	6707 (65.4%)	1765 (17.2%)	1783 (17.4%)	
	70–79	4176 (72.0%)	909 (15.7%)	716 (12.3%)	
Ethnicity					< 0.001
	Caucasian	9007 (70.6%)	1929 (15.1%)	1820 (14.3%)	
	Asian	299 (67.5%)	82 (18.5%)	62 (14.0%)	
	African-American	3573 (58.5%)	1263 (20.7%)	1270 (20.8%)	
	Hispanic	1341 (66.6%)	330 (16.4%)	343 (17.0%)	
	Other/unspecified	254 (55.7%)	95 (20.8%)	107 (23.5%)	
Education					< 0.001
	Less than high school	4987 (60.7%)	1576 (19.2%)	1651 (20.1%)	
	College	5437 (67.9%)	1308 (16.3%)	1257 (15.7%)	
	Higher	3963 (72.8%)	805 (14.8%)	673 (12.4%)	
Type of Job					< 0.001
	Managerial/professional	5435 (70.1%)	1201 (15.5%)	1118 (14.4%)	
	Technical/sales/admin	3749 (65.1%)	1055 (18.3%)	958 (16.6%)	
	Service/labor	2576 (62.1%)	761 (18.3%)	812 (19.6%)	
	Homemaker only	1412 (66.3%)	354 (16.6%)	364 (17.1%)	
Lifestyle Habits					
Alcohol					< 0.001
	Never	1837 (61.0%)	541 (18.0%)	632 (21.0%)	
	Past drinker	2795 (56.6%)	1021 (20.7%)	1119 (22.7%)	
	Current drinker	9721 (71.3%)	2093 (15.4%)	1814 (13.3%)	
Smoking					0.006
	Never	7554 (67.1%)	1919 (17.0%)	1792 (15.9%)	
	Past smoker	5562 (65.2%)	1476 (17.3%)	1487 (17.4%)	
	Current smoker	1166 (68.6%)	259 (15.2%)	274 (16.1%)	
Recreation Physical Activity					< 0.001
(MET-hour)	≥ 0 to < 2	848 (62.6%)	225 (16.6%)	281 (20.8%)	
	≥ 2 to < 8	702 (59.8%)	223 (19.0%)	249 (21.2%)	
	≥ 8 to < 18	733 (67.6%)	187 (17.3%)	164 (15.1%)	
	≥ 18	3017 (76.0%)	509 (12.8%)	444 (11.2%)	
Anthropometric Measures					
Baseline Body Mass Index					< 0.001
(kg/m^2^)	Normal (18.5–24.9)	4379 (91.7%)	288 (6.0%)	108 (2.3%)	
	Overweight (25.0–29.9)	5618 (75.1%)	1126 (15.1%)	735 (9.8%)	
	Obese (≥ 30.0)	3847 (44.5%)	2166 (25.1%)	2628 (30.4%)	
Baseline Waist to Hip Ratio					< 0.001
	Normal (< 0.800)	6869 (84.1%)	798 (9.8%)	498 (6.1%)	
	Overweight (0.800–0.849)	3825 (66.9%)	1068 (18.7%)	825 (14.4%)	
	Obese (≥ 0.850)	3574 (47.1%)	1792 (23.6%)	2223 (29.3%)	
Hormone-Related Factors					
Estrogen-alone					< 0.001
	Never	9915 (66.1%)	2541 (16.9%)	2540 (16.9%)	
	Past	2264 (64.0%)	658 (18.6%)	615 (17.4%)	
	Current	2295 (70.8%)	502 (15.5%)	445 (13.7%)	
Estrogen plus Progestin					< 0.001
	Never	11,966 (64.8%)	3251 (17.6%)	3250 (17.6%)	
	Past	1051 (71.8%)	215 (14.7%)	198 (13.5%)	
	Current	1461 (78.9%)	235 (12.7%)	156 (8.4%)	
Menstrual Cycle Regularity					< 0.001
	No	990 (62.5%)	272 (17.2%)	321 (20.3%)	
	Yes	12,069 (66.9%)	3068 (17.0%)	2898 (16.1%)	
	Sometimes irregular	1306 (65.4%)	335 (16.8%)	355 (17.8%)	
Age at Menopause					< 0.001
	45 or younger	4021 (62.6%)	1154 (18.0%)	1252 (19.5%)	
	46 to 49	4419 (69.4%)	1001 (15.7%)	948 (14.9%)	
	50 to 51	1837 (70.0%)	432 (16.5%)	356 (13.6%)	
	52 or older	3067 (68.7%)	743 (16.6%)	655 (14.7%)	
Parity					< 0.001
	Never pregnant	1403 (65.5%)	371 (17.3%)	367 (17.1%)	
	1	3279 (68.6%)	794 (16.6%)	709 (14.8%)	
	2	3339 (68.3%)	806 (16.5%)	746 (15.3%)	
	3	2182 (65.3%)	575 (17.2%)	586 (17.5%)	
	4	2439 (60.9%)	746 (18.6%)	823 (20.5%)	
	5	1737 (70.7%)	376 (15.3%)	344 (14.0%)	
Hysterectomy					< 0.001
	No	8367 (69.9%)	1906 (15.9%)	1695 (14.2%)	
	Yes	6112 (62.3%)	1793 (18.3%)	1908 (19.4%)	
Medical History					
Diabetes					< 0.001
	No	13,825 (69.8%)	3280 (16.6%)	2698 (13.6%)	
	Yes	650 (33.0%)	419 (21.3%)	899 (45.7%)	
Hypertension					< 0.001
	No	9365 (74.5%)	1787 (14.2%)	1422 (11.3%)	
	Yes	4990 (55.5%)	1882 (20.9%)	2124 (23.6%)	
Cardiovascular Disease					< 0.001
	No	11,382 (67.7%)	2810 (16.7%)	2617 (15.6%)	
	Yes	2179 (60.8%)	667 (18.6%)	740 (20.6%)	
Stroke					< 0.001
	No	14,316 (66.7%)	3628 (16.9%)	3512 (16.4%)	
	Yes	164 (49.8%)	73 (22.2%)	92 (28.0%)	
Liver Disease Ever					0.008
	No	14,187 (66.6%)	3610 (17.0%)	3500 (16.4%)	
	Yes	296 (60.4%)	91 (18.6%)	103 (21.0%)	
Bleeding Problems Ever					0.504
	No	14,142 (66.5%)	3611 (17.0%)	3509 (16.5%)	
	Yes	330 (64.5%)	88 (17.2%)	94 (18.4%)	
History Skin Cancer					< 0.001
	No	13,229 (65.8%)	3474 (17.3%)	3412 (17.0%)	
	Yes	1230 (74.8%)	226 (13.7%)	189 (11.5%)	
Rheumatoid Arthritis Ever					0.010
	Other/do not know	5874 (64.2%)	1638 (17.9%)	1634 (17.9%)	
	Yes	778 (60.3%)	242 (18.7%)	271 (21.0%)	

The time-to-event analytic cohort consisted of 21,789 postmenopausal women. The table shows their baseline demographics, personal habits, and medical history by baseline HOMA-IR. The degree of insulin resistance was categorized as defined by previous studies: Low (HOMA-IR < 1.4), Moderate (1.4 ≤ HOMA-IR < 2.0), and High (HOMA-IR ≥ 2.0) (18–20). Differences in baseline characteristics among HOMA-IR categories were assessed using chi-square tests

CI, confidence interval; HR, hazard ratio; MET, metabolic equivalent of task; HOMA-IR, homeostasis model assessment for insulin resistance

Psoriasis incidence rate was 2.36 cases per 1000 persons per year over the median cumulative follow-up of 9.5 years (interquartile range, 4.5–14.7 years). The majority of the study population was Caucasian (58.6%) or African-American (28.0%) and between 60–69 years old (47.1%). Baseline characteristics by psoriasis incidence are described in Table 2. The average age at first incidence of psoriasis was 74.6 years, occurring on average 8.3 years into the study enrollment.

**Table 2:** The distribution and risk of psoriasis by baseline demographic, personal habits, and medical history

		No psoriasis *n* (%)	Psoriasis *n* (%)	HR (95% CI)	*P* value
Clinical Trials					
Estrogen-Alone Trial					
	Not randomized	11,549 (97.9%)	250 (2.1%)	–-	
	Placebo	2002 (97.4%)	54 (2.6%)	Reference	0.601
	Treated	2081 (97.7%)	50 (2.3%)	0.92 (0.63, 1.35)	0.965
Estrogen + Progestin Trial					
	Not randomized	11,549 (97.9%)	250 (2.1%)	–-	
	Placebo	2764 (97.3%)	76 (2.7%)	Reference	0.904
	Treated	2901 (97.9%)	62 (2.1%)	0.79 (0.56, 1.10)	0.165
Calcium Vitamin D Trial					
	Not randomized	14,046 (97.7%)	332 (2.3%)	–-	
	Placebo	3625 (98.0%)	75 (2.0%)	Reference	
	Treated	3626 (97.7%)	85 (2.3%)	1.12 (0.82, 1.53)	0.473
Dietary Modification Trial					
	Not randomized	15,301 (97.7%)	365 (2.3%)	–-	
	Placebo	2416 (97.6%)	59 (2.4%)	Reference	
	Treated	3580 (98.1%)	68 (1.9%)	0.79 (0.56, 1.12)	0.190
Demographics					
Age					
	50–59	5668 (98.9%)	65 (1.1%)	Reference	
	60–69	9972 (97.2%)	283 (2.8%)	1.18 (0.90, 1.56)	0.234
	70–79	5657 (97.5%)	144 (2.5%)	0.84 (0.62, 1.13)	0.253
Ethnicity					
	Caucasian	12,399 (97.2%)	357 (2.8%)	Reference	
	Asian	434 (98.0%)	9 (2.0%)	0.83 (0.43, 1.61)	0.579
	African-American	6021 (98.6%)	85 (1.4%)	0.67 (0.53, 0.85)	0.001
	Hispanic	1980 (98.3%)	34 (1.7%)	0.93 (0.65, 1.32)	0.681
	Other/unspecified	449 (98.5%)	7 (1.5%)	0.76 (0.36, 1.60)	0.466
Education					
	Less than high school	8048 (98.0%)	166 (2.0%)	Reference	
	College	7818 (97.7%)	184 (2.3%)	1.11 (0.90, 1.36)	0.346
	Higher	5304 (97.5%)	137 (2.5%)	1.17 (0.93, 1.46)	0.178
Type of Job					
	Managerial/professional	7567 (97.6%)	187 (2.4%)	Reference	
	Technical/sales/admin	5648 (98.0%)	114 (2.0%)	0.84 (0.67, 1.06)	0.152
	Service/labor	4059 (97.8%)	90 (2.2%)	0.96 (0.75, 1.24)	0.772
	Homemaker only	2076 (97.5%)	54 (2.5%)	1.02 (0.75, 1.38)	0.910
Lifestyle Habits					
Alcohol					
	Never	2963 (98.4%)	47 (1.6%)	Reference	
	Past drinker	4829 (97.9%)	106 (2.1%)	1.45 (1.03, 2.04)	0.034
	Current drinker	13,294 (97.5%)	334 (2.5%)	1.51 (1.12, 2.05)	0.008
Smoking					
	Never	11,057 (98.2%)	208 (1.8%)	Reference	
	Past smoker	8294 (97.3%)	231 (2.7%)	1.52 (1.26, 1.83)	< 0.001
	Current smoker	1653 (97.3%)	46 (2.7%)	1.89 (1.38, 2.61)	< 0.001
Recreation Physical Activity					
(MET-hour)	≥ 0 to < 2	1310 (96.8%)	44 (3.2%)	Reference	
	≥ 2 to < 8	1147 (97.7%)	27 (2.3%)	0.70 (0.44, 1.14)	0.150
	≥ 8 to < 18	1064 (98.2%)	20 (1.8%)	0.55 (0.32, 0.93)	0.026
	≥ 18	3878 (97.7%)	92 (2.3%)	0.66 (0.46, 0.94)	0.021
Anthropometric Measures					
Baseline Body Mass Index					
(kg/m2)	Normal (18.5–24.9)	4663 (97.7%)	112 (2.3%)	Reference	
	Overweight (25.0–29.9)	7321 (97.9%)	158 (2.1%)	0.94 (0.74, 1.20)	0.606
	Obese (≥ 30.0)	8437 (97.6%)	204 (2.4%)	1.13 (0.90, 1.43)	0.288
Baseline Waist to Hip Ratio					
	Normal (< 0.800)	7998 (98.0%)	167 (2.0%)	Reference	
	Overweight (0.800–0.849)	5592 (97.8%)	126 (2.2%)	1.09 (0.87, 1.38)	0.458
	Obese (≥ 0.850)	7395 (97.4%)	194 (2.6%)	1.28 (1.04, 1.57)	0.020
Hormone-Related Factors					
Estrogen-alone					
	Never	14,676 (97.9%)	320 (2.1%)	Reference	
	Past	3443 (97.3%)	94 (2.7%)	1.16 (0.92, 1.46)	0.200
	Current	3164 (97.6%)	78 (2.4%)	1.26 (0.98, 1.61)	0.068
Estrogen plus Progestin					
	Never	18,036 (97.7%)	431 (2.3%)	Reference	
	Past	1433 (97.9%)	31 (2.1%)	0.96 (0.67, 1.38)	0.828
	Current	1822 (98.4%)	30 (1.6%)	0.82 (0.56, 1.18)	0.285
Menstrual Cycle Regularity					
	No	1542 (97.4%)	41 (2.6%)	Reference	
	Yes	17,623 (97.7%)	412 (2.3%)	0.85 (0.61, 1.17)	0.308
	Sometimes irregular	1959 (98.1%)	37 (1.9%)	0.70 (0.45, 1.09)	0.116
Age at Menopause					
	45 or younger	6289 (97.9%)	138 (2.1%)	Reference	
	46 to 49	6229 (97.8%)	139 (2.2%)	1.01 (0.80, 1.28)	0.930
	50 to 51	2562 (97.6%)	63 (2.4%)	1.07 (0.79, 1.44)	0.666
	52 or older	4358 (97.6%)	107 (2.4%)	0.99 (0.77, 1.28)	0.959
Parity					
	Never pregnant	2099 (98.0%)	42 (2.0%)	Reference	
	1	4656 (97.4%)	126 (2.6%)	1.29 (0.91, 1.83)	0.152
	2	4783 (97.8%)	108 (2.2%)	1.04 (0.73, 1.49)	0.830
	3	3262 (97.6%)	81 (2.4%)	1.12 (0.77, 1.63)	0.542
	4	3932 (98.1%)	76 (1.9%)	0.91 (0.62, 1.32)	0.616
	5	2403 (97.8%)	54 (2.2%)	1.05 (0.70, 1.57)	0.827
Hysterectomy					
	No	11,714 (97.9%)	254 (2.1%)	Reference	
	Yes	9575 (97.6%)	238 (2.4%)	1.20 (1.00, 1.43)	0.046
Medical History					
Diabetes					
	No	19,354 (97.7%)	449 (2.3%)	Reference	
	Yes	1925 (97.8%)	43 (2.2%)	1.05 (0.77, 1.43)	0.773
Hypertension					
	No	12,279 (97.7%)	295 (2.3%)	Reference	
	Yes	8801 (97.8%)	195 (2.2%)	0.90 (0.75, 1.08)	0.274
Cardiovascular Disease					
	No	16,446 (97.8%)	363 (2.2%)	Reference	
	Yes	3492 (97.4%)	94 (2.6%)	1.14 (0.91, 1.43)	0.246
Stroke					
	No	20,970 (97.7%)	486 (2.3%)	Reference	
	Yes	323 (98.2%)	6 (1.8%)	0.89 (0.40, 1.99)	0.773
Liver Disease Ever					
	No	20,819 (97.8%)	478 (2.2%)	Reference	
	Yes	476 (97.1%)	14 (2.9%)	1.29 (0.76, 2.20)	0.341
Bleeding Problems Ever					
	No	20,783 (97.7%)	479 (2.3%)	Reference	
	Yes	499 (97.5%)	13 (2.5%)	1.16 (0.67, 2.01)	0.602
History Skin Cancer					
	No	19,685 (97.9%)	430 (2.1%)	Reference	
	Yes	1583 (96.2%)	62 (3.8%)	1.44 (1.10, 1.88)	0.007
Rheumatoid Arthritis Ever					
	Other/do not know	8918 (97.5%)	228 (2.5%)	Reference	
	Yes	1253 (97.1%)	38 (2.9%)	1.26 (0.89, 1.78)	0.186

The time-to-event analytic cohort consisted of 21,789 postmenopausal women. The table shows their baseline demographics, personal habits, and medical history by psoriasis incidence over the 21-years cumulative follow-up period. Hazard rates and 95% confidence intervals were stratified on age (10-year intervals), on WHI component (Clinical Trial or Observational Study), and on randomization status within each of the WHI clinical trials (Diet Modification, Hormone Therapy, Calcium and Vitamin-D Trial)

CI, confidence interval; HR, hazard ratio; MET, metabolic equivalent of task; HOMA-IR, homeostasis model assessment for insulin resistance

**Fig. 1:**
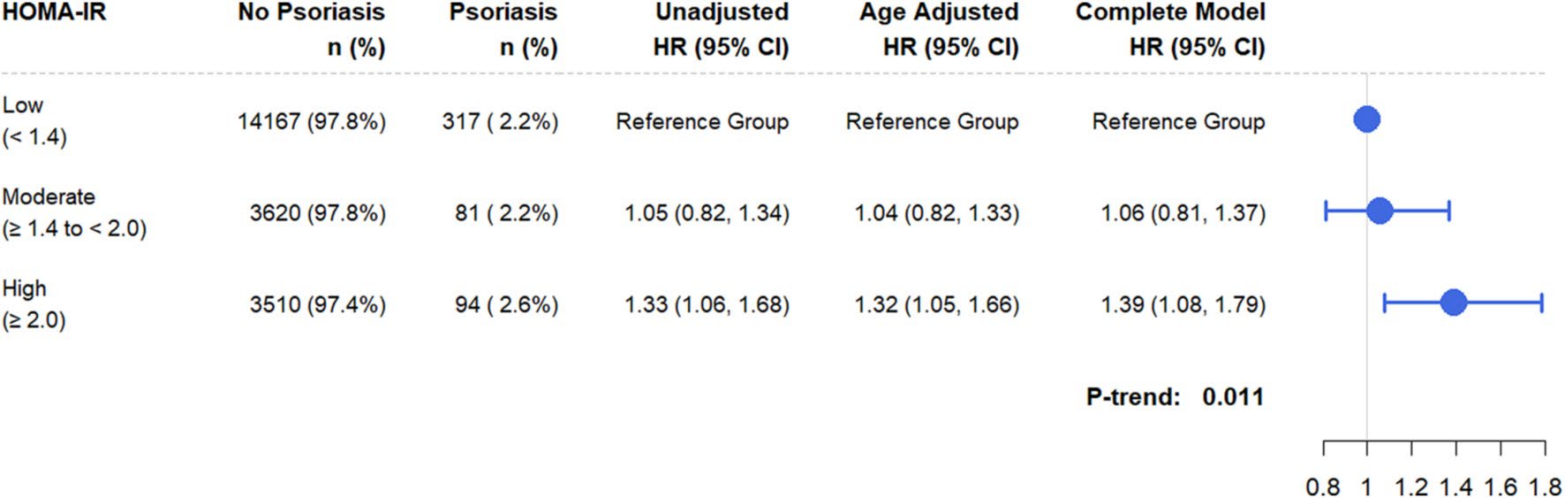
Psoriasis Time-to-Event analysis by HOMA-IR. Table shows psoriasis distribution by HOMA-IR and the resulting hazard ratios (HR’s) and confidence intervals (CI’s) from cox-regression analysis: unadjusted, age adjusted, and the complete model. The hazard rates from the complete model were stratified on age (10-year intervals), on WHI component (Clinical or Observational Study), and on randomization status within each of the WHI clinical trials (Diet Modification, Hormone Therapy, Calcium and Vitamin-D Trial). In addition, the following baseline characteristics were included in the complete model: ethnicity (Caucasian, Asian, African-American, Hispanic, Other/Unspecified), continuous waist–hip-ratio, smoking (non-smoker, past-smoker, current-smoker), and alcohol habits (non-drinker, past-drinker, current drinker). The forest plot to the right of the table reflects the hazard ratios from the complete model and show that “high HOMA-IR” group had higher risk for psoriasis compared to the “low HOMA-IR” group

**Fig. 2:**
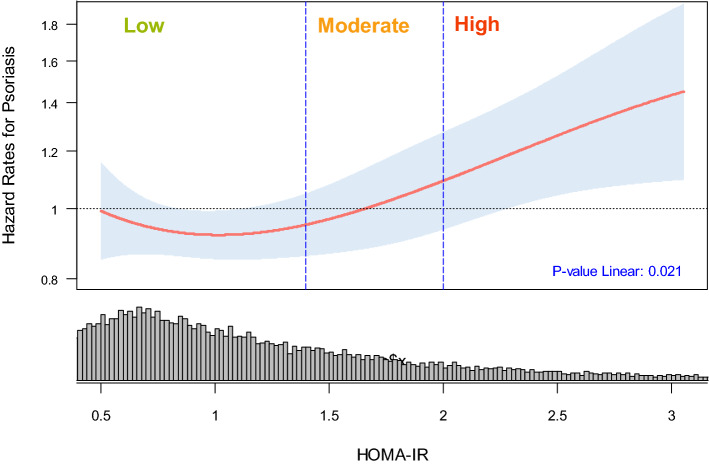
Psoriasis partial hazard (*y*-axis) by HOMA-IR (x-axis) as a continuous variable. The red line and the 95% confidence interval (blue shading) were predicted using a spline term on HOMA-IR with four degrees of freedom. The histogram at the bottom shows the distribution of HOMA-IR within the analytic cohort. Degree of insulin resistance was categorized as defined by previous studies: Low (HOMA-IR < 1.4), Moderate (1.4 ≤ HOMA-IR < 2.0), and High (HOMA-IR ≥ 2.0) [18–20]

The multivariable analysis adjusts for ethnicity, continuous waist–hip-ratio, smoking and alcohol habits, and was stratified on age (10-year interval), on WHI components (Clinical Trial or Observational Study), and on randomization status within each of the WHI clinical trials (Diet Modification, Hormone Therapy, Calcium and Vitamin-D Trial). Comparing psoriasis risk in HOMA-IR high (≥ 2.0) versus low (< 1.4), the estimated hazard ratio was 1.39 (95% CI 1.08–1.79, *P*-trend: 0.011) (Fig. 1). Spline-based partial hazard estimates for psoriasis was mostly linear for continuous values of HOMA-IR (*P*-value Linear: 0.016) (Fig. 2).

**Fig. 3:**
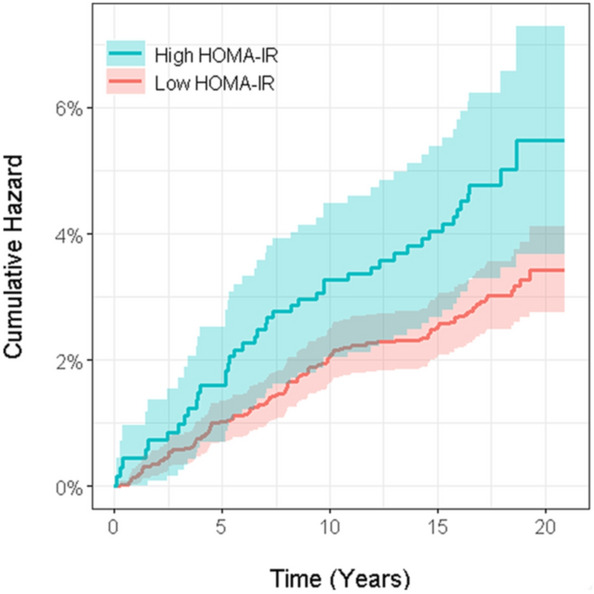
Psoriasis Cumulative Hazard Curve by HOMA-IR among non-smoking women. Over the 21-year follow-up period (*x*-axis), the psoriasis cumulative hazard (*y*-axis) was steadily and consistently higher in the High HOMA-IR group (Blue) compared to the Low HOMA-IR group (Red) among non-smoking women. The respective colored shading represents the 95% confidence interval

In analyses stratified by smoking (a major confounder for psoriasis), no interaction between smoking and HOMA-IR was detected (*P*-interaction = 0.472). The Kaplan–Meier curve (cumulative hazard over time) shows that among non-smoking women, the risk for psoriasis was steadily and consistently higher in women with high HOMA-IR (≥ 2.0) compared to low (< 1.4) over the 21-year cumulative follow-up period (Fig. 3).

In sensitivity analyses excluding women with potentially confounding factors [such as baseline-treated diabetes (*n* = 1968), a history of hypertension (*n* = 8996), cardiovascular disease (*n* = 3586), rheumatoid arthritis (*n* = 1291), or non-melanoma skin cancer (*n* = 1645)], higher insulin resistance remained significantly associated with greater psoriasis incidence.

## Discussion

Higher baseline insulin resistance assessed by the updated HOMA2 version 2.2.3 was significantly associated with greater incidence of psoriasis in postmenopausal women. Therefore, while other epidemiological studies suggest that psoriasis precedes type II diabetes [12, 13], the risk of psoriasis itself may be attributed to a pre-diabetic condition, with high insulin resistance assessed using HOMA-IR. While the exact mechanisms remain unclear, several theories have been proposed for the pathophysiologic link between psoriasis and insulin resistance. This may be in part due to shared genetic regions of susceptibility between psoriasis and diabetes [23].

While the exact mechanisms that link psoriasis and insulin resistance have not been described, immune dysregulation has been reported to play a key role in both conditions [24]. T-helper cell 1 (Th1) signaling pathway have been implicated in both psoriasis and insulin resistance. A shift in the macrophage population towards more pro-inflammatory M1 than anti-inflammatory M2 has been implicated in both conditions [25]. Cytokine production through promotion of NF-κB (nuclear factor kappa light chain enhancer of activated B cells) transcription factor signaling, which may also contribute to macrophage activation, has also been implicated in both conditions [26, 27]. Overproduction of pro-inflammatory cytokines such as tumor necrosis factor alpha (TNFα) and interleukin (IL)-1β is associated with insulin resistance [28–31]. IL-1β blocks insulin-dependent differentiation of keratinocytes and drives keratinocyte proliferation, both of which are hallmarks of psoriasis pathogenesis [32]. TNFα is also directly implicated in psoriasis pathogenesis and TNFα inhibitors are recommended as a monotherapy treatment option for adults with moderate-to-severe psoriasis [4]. These findings, combined with results from the time-to-event analysis, support the notion that insulin resistance is associated with an increased risk of psoriasis and can precede skin pathology.

Previous studies have also identified a possible relationship between psoriasis and metabolic syndrome, which is associated with insulin resistance and psoriasis severity. For example, patients with metabolic syndrome had significantly higher Psoriasis Area Severity Index (PASI) scores compared to those without metabolic syndrome. Furthermore, in patients without metabolic syndrome, HOMA-IR significantly correlated with PASI score [33]. This is also consistent with a large meta-analysis of observational studies in which patients with more severe psoriasis had greater odds of metabolic syndrome [12]. Our findings suggest that insulin resistance might be explored as an additional therapeutic target in psoriasis patients, especially in those with concomitant diabetes.

Anti-diabetic agents have anti-inflammatory properties in the setting of insulin resistance, obesity, and heart disease [34]. Optimistic reports (case reports, case series, and small clinical trials) suggest thiazolidinediones (TZDs) may provide clinical benefits for psoriasis through a decrease of cytokine production including TNFα; however, TZDs have not been consistently shown to improve disease severity [35]. Metformin may promote macrophage activation toward the M1 phenotype through the AMPK/NFκB pathway [36], but other data suggest it could decrease inflammatory cytokines [37]. Human cohort data demonstrated no effect of metformin on morbidity and mortality in psoriasis patients with type 2 diabetes [38].

### Strengths and limitations

While the accuracy of ICD-9-CM diagnostic codes has a positive predictive value of 89% (95% CI 79–95%) in the Northern California population, a limitation of our study is the use of ICD-9-CM diagnostic codes from Medicare claims reports to identify psoriasis cases [16]. Although hyperinsulinemic euglycemic clamp (HEC) is considered the gold standard for evaluating insulin sensitivity, estimates derived from HOMA-IR strongly correlate with HEC [6–8, 39]. Lastly, our cohort is limited to postmenopausal women, therefore, limiting the generalizability of these findings.

Compared to previous studies (case–control studies with *n* < 200), a key strength of our analysis is the use of a large study cohort, which provides for a more stable and robust multivariable model that accounts for key confounders for psoriasis such as obesity and smoking habits. More importantly, our analysis takes into consideration the time variable and indicates the temporal sequence between exposure and outcome compared to previous case–control studies. Furthermore, most of the previous studies on HOMA-IR used a simple mathematical approximation while we used the updated HOMA2 version 2.2.3 [17]. Whereas BMI and WHR are subject to change in aging women, insulin resistance by HOMA-IR provides a metabolic functional measurement independent of anatomic measurements.

## Conclusion

Psoriasis is a systemic inflammatory skin disease associated with significant comorbidities. In postmenopausal women, higher baseline insulin resistance assessed by HOMA-IR was significantly associated with an increased risk of psoriasis during a cumulative 21-year follow-up. While previous studies suggested that insulin resistance is a sequela of psoriasis, results from this time-to-event analysis indicate that insulin resistance can precede and is associated with an increased risk of psoriasis. Findings may warrant implementing lifestyle changes such as diet and exercise to reduce insulin resistance, which may ultimately improve or reduce the risk of psoriasis. Further research is warranted to investigate the underlying pathophysiology linking psoriasis and insulin resistance as well as the potential role of hypoglycemic agents in psoriasis management.

## Supplementary Information

Below is the link to the electronic supplementary material.

Supplementary file1 (DOCX 855 KB)
