# Supplementary material for: Association between baseline insulin resistance and psoriasis incidence: the Women’s Health Initiative
Source: Arch Dermatol Res. 2021 Nov 24;314(9):869–80. doi: 10.1007/s00403-021-02298-9 (PMC9512862; doi:10.1007/s00403-021-02298-9)

**Figure S1** – The boxplots below show that the baseline insulin and glucose were mostly similar across the different testing version IDs among Caucasian women.

**
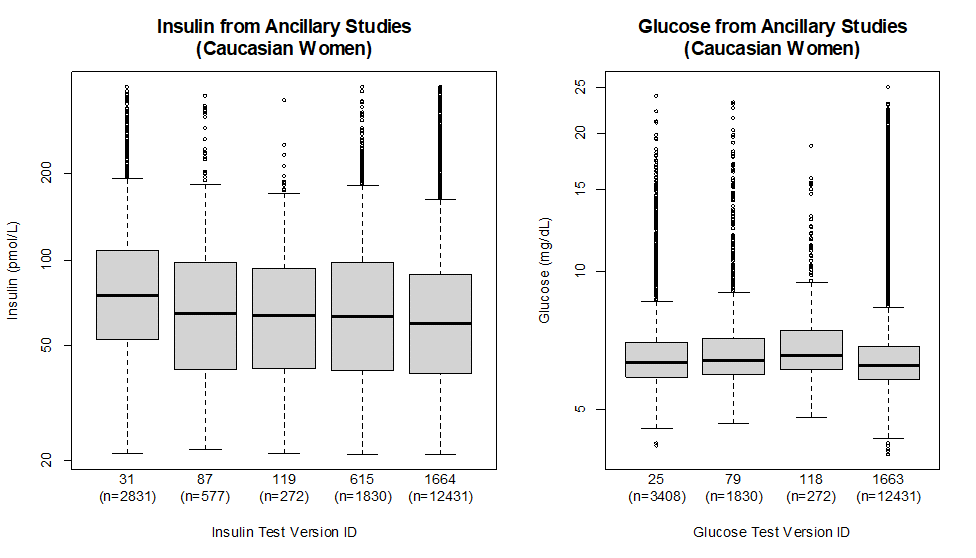
**

**Figure S2** – shows the association between HOMA-IR with [A] Body Mass Index (BMI) and with [B] Waist Hip Ratio (WHR). The correlation coefficient (R) was calculated using Pearson correlation. The figure shows that there is a subgroup of women who were obese (BMI > 30 kg/m^2^ or WHR > 0.85), but had low insulin resistance and vice versa. Insulin resistance provides a metabolic functional measurement independent of anatomic information from BMI and WHR, which are subject to change in aging women.

[A] BMI and HOMA-IR


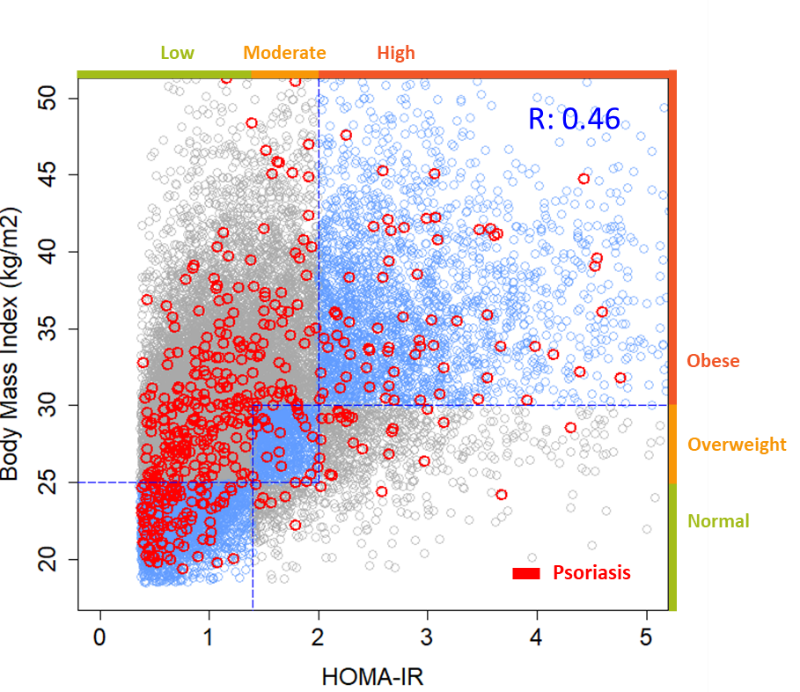


[B] WHR and HOMA-IR


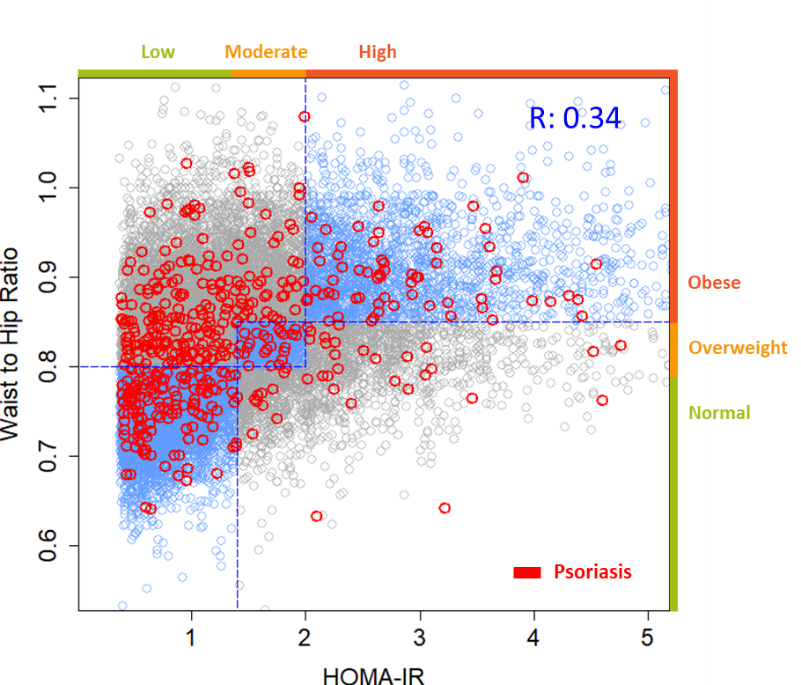

Supplement: Supplementary file 1 — Supplementary file1 (DOCX 855 KB) [file 403_2021_2298_MOESM1_ESM.docx]
